# Radical prostatectomy *versus* high intensity focused ultrasound for localized prostate cancer: a matched pair comparison

**DOI:** 10.1186/2050-5736-3-S1-O56

**Published:** 2015-06-30

**Authors:** Albert Gelet, Sebastien Crouzet, Olivier Rouviere, Jean-Yves Chapelon, Murielle Rabilloud

**Affiliations:** 1 Edouard Herriot Hospital, Lyon, France; 2 Hospices Civils de Lyon, Lyon, France; 3 Inserm, Lyon, France

## Background/introduction

Radical prostatectomy is the gold standard treatment for localized prostate cancer. HIFU is a treatment option with promising outcomes. No randomized study is available to compare those techniques. The goal of the study was to evaluate the oncologic outcome of patients treated with HIFU and radical prostatectomy by using a matched pair analysis to compare the 2 groups.

## Methods

A total of 710 patients treated between 2000 and 2005 were prospectively followed in our institutional database and matched to a 1:1 basis following know prognostic variables: prostate-specific antigen (PSA) level, Gleason score, and clinical stage. After matching, 588 patients (294 in each group) were further analysed. The starts of salvage external beam radiotherapy (S-EBRT) or definitive palliative androgen deprivation therapy (ADT) were primary endpoints. Other endpoints were overall, cancer specific and metastasis free survival rates: The survival rates were calculated with Kaplan-Meier estimate.

## Results and conclusions

The seven years S-EBRT free survival rate was significantly lower after HIFU than after RP (62% *versus* 78%, p=0.001). The palliative androgen deprivation free rate at nine years was not significantly different between HIFU and RP (86% *versus* 87%, p=0.271). At nine years the overall, cancer specific and metastasis free survival rates were similar: 89%, 97%, 94 % and 89%, 97% and 97% for HIFU and RP respectively (p=0.186, 0.312, 0.107). Matched pair comparison of HIFU and RP has shown a higher rate of S-EBRT for HIFU. At 9 years, the rate of patients who need palliative ADT, the overall cancer specific and metastasis free survival rates were not significantly different between HIFU and RP.

